# Supplementary material for: Comparative genomics provides new insights into the diversity, physiology, and sexuality of the only industrially exploited tremellomycete: Phaffia rhodozyma
Source: BMC Genomics. 2016 Nov 9;17:901. doi: 10.1186/s12864-016-3244-7 (PMC5103461; doi:10.1186/s12864-016-3244-7)
Supplement: Additional file 6: — List of orphan genes with links to PFAM (related to Additional file 1: Table S1). (ZIP 1428 kb) [file 12864_2016_3244_MOESM6_ESM.zip › PFAM_UNIQUE/G01756_P.html]

#                                                                            --- full sequence --- -------------- this domain -------------   hmm coord   ali coord   env coord
  
# target name        accession   tlen query name           accession   qlen   E-value  score  bias   #  of  c-Evalue  i-Evalue  score  bias  from    to  from    to  from    to  acc description of target
  
#------------------- ---------- ----- -------------------- ---------- ----- --------- ------ ----- --- --- --------- --------- ------ ----- ----- ----- ----- ----- ----- ----- ---- ---------------------
  
G01756\_P             -            246 DctM                 PF06808.7    416    0.0095   13.2   0.1   1   1   1.1e-05     0.013   12.8   0.1   133   203    27    98    24   127 0.80 - DctM-like transporters  
G01756\_P             -            246 DUF4199              PF13858.1    163     0.023   13.3   0.2   1   1   2.2e-05     0.033   12.8   0.2   130   162    61    93    28    94 0.71 - Protein of unknown function (DUF4199)  
G01756\_P             -            246 Herpes\_gE            PF02480.11   439     0.038   10.9   0.0   1   1     9e-05     0.049   10.5   0.0   327   387    42   104    30   133 0.80 - Alphaherpesvirus glycoprotein E  
G01756\_P             -            246 Syndecan             PF01034.15    64     0.011   14.0   0.0   1   1   2.2e-05     0.022   13.1   0.0    11    53    65   112    55   117 0.68 - Syndecan domain  
G01756\_P             -            246 Ustilago\_mating      PF05722.7    286       6.8    4.6   8.6   1   2   0.00018       1.1    7.2   4.6   122   168    19    65     7    68 0.90 - Ustilago B locus mating-type protein  
G01756\_P             -            246 Ustilago\_mating      PF05722.7    286       6.8    4.6   8.6   2   2      0.17     1e+03   -2.6   0.1   159   176   206   223   183   226 0.72 - Ustilago B locus mating-type protein
